# Executives’ IT background and corporate digital technology innovation: Evidence from Chinese microenterprises

**DOI:** 10.1371/journal.pone.0320844

**Published:** 2025-04-28

**Authors:** Shuang Cao, Yingjue Wu

**Affiliations:** School of International business, Hainan University, Haikou, China; Islamic Azad University Urmia Branch, IRAN, ISLAMIC REPUBLIC OF

## Abstract

This study examines how executives’ IT backgrounds affect corporate digital technology innovation, using panel data from listed companies between 2011 and 2021. Findings indicate that executives’ IT backgrounds significantly enhance corporate digital technology innovation, particularly in digital technology invention patents, where the effect is especially pronounced, and the results are robust. Mechanistic analysis suggests managerial myopia and executives’ attention to digital technology are key factors that allow executives’ IT backgrounds to promote corporate digital technology innovation. Further analysis shows executives’ IT backgrounds have a greater promotional effect on digital technology innovation in state-owned enterprises, non-IT sectors, firms with influential executives, and organizations with long-serving executives. Economic analysis reveals that executives’ IT backgrounds can improve corporate financial performance by enhancing digital technology innovation. This paper highlights the importance of executive IT human capital and provides insights into government and corporate talent recruitment policies.

## 1. Introduction

Innovation is crucial for maintaining a business’s competitive edge and is the primary driver of national economic growth [1]. The rise of digital technologies, including artificial intelligence, blockchain, cloud computing, big data, and the Internet of Things, marks a new era of technological revolution and industrial change [2]. Fostering the digital economy is now a strategic move to capture new opportunities. Digital technology innovation, the backbone of the digital economy, is crucial for the next competition phase [3]. The 2022 report from the 20th National Congress of the Communist Party of China set the goal to ‘speed up the development of a cyber powerhouse and digital China.’ Over the years, China has consistently advanced its digital infrastructure and made significant strides in digital technology innovation. The ‘Digital China Development Report (2021)’ by the Cyberspace Administration of China shows a 60% rise in China’s PCT international patent applications in the information sector 2021 from 2017, surpassing one-third of the global total.

In this context, digital technology innovation has attracted unparalleled attention. As the primary driver of China’s digital economy, digital technology innovation offers traditional enterprises a vital path to digital transformation and high-quality growth. It is crucial in boosting China’s digital economy’s competitiveness and global influence [4]. However, many enterprises are not motivated to innovate or pursue digital transformation. On one hand, most enterprises are in the preliminary stages of applying digital technologies, revealing an apparent deficiency in their digital foundations and capabilities [5]. Digital technology innovation demands significant upfront investments and a willingness to accept the risks of innovation’s uncertainties [6]. On the other hand, progress in digital technology innovation heavily relies on information technology talent [7]. Meanwhile, senior executives play a pivotal role in guiding corporate strategy and its execution, being central to the company’s development [8]. However, in many enterprises, senior executives often lack a background in information technology [9].

A small body of literature has begun to focus on the pathways to achieving digital technology innovation; digital technology innovation relies on knowledge acquisition and information exchange with other market entities within the industry chain [10,6]. However, the impact of executives’ IT backgrounds on digital technology innovation still needs to be empirically explored, presenting an opportunity for this study to contribute. Exploring how executives’ IT backgrounds directly impact corporate digital innovation and the underlying mechanisms is crucial for enhancing digital technology innovation capabilities, developing a digital technology innovation system, and advancing digital transformation. This paper not only enriches theoretical frameworks but also provides practical applications.

The innovations of this study are in the following aspects. (1) Approaching from the perspective of executive characteristics, this study focuses on the IT backgrounds of executives and confirms that such a trait significantly enhances corporate digital technology innovation, highlighting the crucial role of leaders’ background characteristics in digital technology innovation and providing valuable insights for the field. (2) This study delves into the mechanisms through which executives’ information technology backgrounds influence corporate digital technology innovation, examining the roles of managerial myopia and executives’ attention to digital technology. It contributes a new theoretical framework for future research in this domain. (3) From the perspective of heterogeneity analysis, it reveals the reinforcing role of property rights, industry types, executive power, and tenure in the process of executives’ information technology backgrounds influence on corporate digital technology innovation, which can provide new perspectives for future research into corporate innovation incentives and the refinement of corporate governance strategies. In conclusion, this paper expands the existing literature on executives’ IT backgrounds and corporate digital technology innovation. It aids firms in selecting IT talent wisely during the early stages of digital transformation, maximizing the role of IT professionals in promoting digital innovation.

## 2. Theoretical background and research hypotheses

### 2.1 Theoretical background

Enterprises, the fundamental micro-units of economic activity, play a pivotal role in driving innovations in digital technology. Current research exhibits confusion between digital technology innovation and digital transformation concepts. Digital technology innovation and digital transformation represent two separate concepts. Digital technology innovation involves enterprises developing new products, processes, and organizational and business models, leveraging digital technology [11,12]. In this definition, digital technology encompasses technologies and their integrations related to information, computation, communication, and connectivity. This includes artificial intelligence, big data, cloud computing, and blockchain [13]. Innovation refers to the process of innovation and its outcomes, often measured in patent output in most studies [14,15]. In contrast, digital transformation focuses on applying digital technology to innovate products, enhance processes, restructure organizations, and develop business models [16,17]. The success of digital transformation hinges on digital technology innovation.

Upper Echelons Theory posits that corporate executives’ traits, including cognitive structures and values, significantly influence decision-making and strategic choices within an organization [18]. Furthermore, executives’ educational and professional backgrounds influence their cognitive structures and values. Numerous studies have explored the economic effects of executives’ experiences from diverse perspectives. Liu et al. (2020) [19] findings suggest that firms with more executives with financial backgrounds engage in less innovation than firms with fewer executives with financial backgrounds. Islam and Zein (2020) [20] found that Inventor CEOs substantially boost corporate R&D investment, innovation output, and efficiency. Quan et al. (2021) [21] document a positive association between CEO foreign experience and corporate green innovation.

As information technology’s role in corporate development grows more apparent, more scholars are studying how executives’ IT backgrounds affect business performance. Bassellier et al. (2003) [22] indicate that CEOs with an IT background are likelier to adopt information technology and encourage its use among employees. Lim et al. (2013) [23] suggest that executives with IT-related education or experience contribute to enhancing corporate reputation. Information technology is fundamental to a high-quality information environment [24], prompting some scholars to explore how executives’ IT backgrounds can improve corporate information environments [25].

Recent research has separately assessed how factors like corporate risk perception [26,27], executive characteristics [28,29], CEOs’ diverse professional backgrounds [30,31], technological investments [32], market competition [33,34], and business models influence digital transformation [35,36]. Existing literature on IT background and corporate digital technology innovation primarily examines the impact of individual traits of executives in specific positions on digital technology innovation, such as the CEO’s IT background [37], the role of female executives [38], executive cognition [39], educational level [40], and digital knowledge [7]. However, such studies tend to generalize the scope of executives, failing to clearly distinguish executives’ specific cognitive and skill requirements in the decision-making and execution of digital technology innovation. As a result, it is not easy to elucidate the role mechanisms of executives in digital technology innovation. There is currently a lack of in-depth research on whether executives’ IT backgrounds effectively drive digital technology innovation. Therefore, the relationship between executives’ information technology backgrounds and digital technology innovation needs more evidence to support it.

### 2.2 Research hypotheses

#### 2.2.1 Executives’ IT background and corporate digital technology innovation.

Digital technology innovation seeks to boost enterprise productivity by lowering internal control costs [41], enhancing investment decision quality [42], and improving asset efficiency and labor resource allocation [43,44]. Digital technology innovations, characterized by long ROI periods and high uncertainty, demand significant investments in capital, technology, equipment, and particularly IT talent [6]. High entry barriers, costs, and risks deter many firms from pursuing digital technology innovation [7]. Executives with IT backgrounds are more likely to drive digital technology innovation than their counterparts without such expertise. First, executives with IT backgrounds are inclined to champion digital technology innovation because they grasp IT system operations and stay current with industry developments, giving them a clearer understanding of the risks and benefits of digital investments [45]. Second, executives’ expertise in digital technologies facilitates the creation of channels that enable enterprises to acquire digital talent and technology quickly and cost-effectively [46], thus boosting the company’s innovation in digital technology. Third, executives with IT backgrounds recognize the enduring significance of digital technology innovation in business development [47]. Their deep understanding of digital technologies’ potential value motivates them to prioritize digital technology innovation at a strategic corporate level. Based on the above analysis, this paper proposes the following hypothesis:

**H1**: Executives’ IT background significantly enhances corporate digital technology innovation.

#### 2.2.2 Executives’ IT background, managerial myopia, and corporate digital technology innovation.

Managerial myopia, linked to managers’ time perception [48,49], influences their behaviors and strategic choices, subsequently affecting organizational actions and outcomes [18,50,51]. Accordingly, the upper echelons theory indicates that managerial myopia affects corporate investment behavior. Digital technology innovation, characterized by significant inputs, high risks, high uncertainty, and long investment cycles, often does not provide immediate economic returns [52,53]. Consequently, myopic managers often prefer investments with shorter terms and higher returns [54–56]. Executives with IT backgrounds can somewhat mitigate managerial myopia, thus impacting the firm’s digital technology innovation [57]. On the one hand, digital technology innovation aims to achieve technological breakthroughs, requiring sustained long-term investment in research and development [16]. Suppose corporate management focuses on short-term profit maximization and employs a short-term transformation strategy. In that case, this approach might constrain the depth and breadth of digital technology innovation, thus impeding breakthroughs and transformations in digital technology [58]. Executives with a background in information technology possess a deep understanding of a company’s business model and digitization level, thanks to their expertise in both technical and managerial roles [46]. Their insight enables them to establish realistic timelines for digital technological innovations, mitigating short-sightedness in management. On the other hand, executives with IT backgrounds assess the comprehensive impact of digital technology innovations rather than relying solely on short-term performance indicators [59], helping them manage the pressures of short-term performance dips effectively. This comprehensive approach prevents the stagnation of digital technology innovations and effectively reduces managerial myopia. Based on the above analysis, this paper proposes the following hypothesis:

**H2**: Executives’ IT background will promote corporate digital technology innovation by alleviating managerial myopia.

#### 2.2.3 Executives’ IT background, executives’ attention to digital technology, and corporate digital technology innovation.

The attention-based view of the upper echelon suggests that in a complex decision-making environment, where information is abundant, corporate executives’ limited rationality, energy, and time make their attention a scarce resource [60]. As diverse external information reaches managers, they selectively focus their limited attention on information that aligns with their needs and goals, evaluating potential further actions [61]. Moreover, the attention-based view indicates that allocating executives’ attention significantly influences corporate behavior, with only the matters that engage their attention being integrated into decision-making processes and affecting corporate actions [62]. Executives’ attention to digital technology is critical to the digital technology innovation process within enterprises, and those with an IT background can further strengthen this focus. On one hand, digital technology innovation is an exploratory activity with uncertain investment outcomes [6]. Given the scarcity of executive attention, how executives focus on high-risk digital technology projects critically affects their strategic decision-making and implementation [63]. To reduce the risks associated with investment decisions in digital technology innovation, IT-savvy executives boost the digital focus of other executives through targeted training [64]. This approach equips executives with indirect experience in digital investments and reduces decision-making uncertainty. On the other hand, executives with information technology backgrounds, by participating in corporate decision-making, emphasize the importance of digital technology innovation among executives [7]. Management’s perception of the importance of digital technology innovation influences the reallocation of innovation resources within the enterprise, demonstrated by increased funding allocation, ensuring continuous and stable investment, ultimately leading to enhanced levels of digital technology innovation within the enterprise [65]. Based on the above analysis, this paper proposes the following hypothesis:

**H3**: Executives’ IT background will promote corporate digital technology innovation by enhancing executives’ attention to digital technology.

#### 2.2.4 Executives’ IT background, digital technology innovation, and corporate financial performance.

Executives with an IT background typically have a strong understanding of digital technology trends, which enables them to identify and introduce cutting-edge technologies like artificial intelligence, big data, automation, and cloud computing, creating innovation opportunities for the company. These technologies optimize internal processes, improve operational efficiency, and enhance the quality of products and services, ultimately boosting customer satisfaction and experience. During digital transformation, executives’ IT expertise enables them to integrate data analytics tools, analyze big data, and help the company accurately understand market demands and customer behavior, leading to more informed decisions. This data-driven approach improves marketing efficiency, increases revenue, and reduces costs, enhancing the company’s financial performance. Moreover, executives’ IT background helps the company retain a competitive edge. As digital technologies evolve, executives with IT expertise can help the company adapt quickly to new technologies and avoid falling behind in the industry’s technological revolution. For example, when applying cutting-edge technologies like cloud computing, the Internet of Things, and blockchain, executives can swiftly drive technology implementation, gaining a market advantage and securing long-term financial returns. In conclusion, executives’ IT background drives digital technology innovation, optimizes business operations, enhances competitiveness, and improves financial performance.

**H4**: Executives’ IT backgrounds enhance corporate financial performance by promoting digital technology innovation.

**Fig 1:**
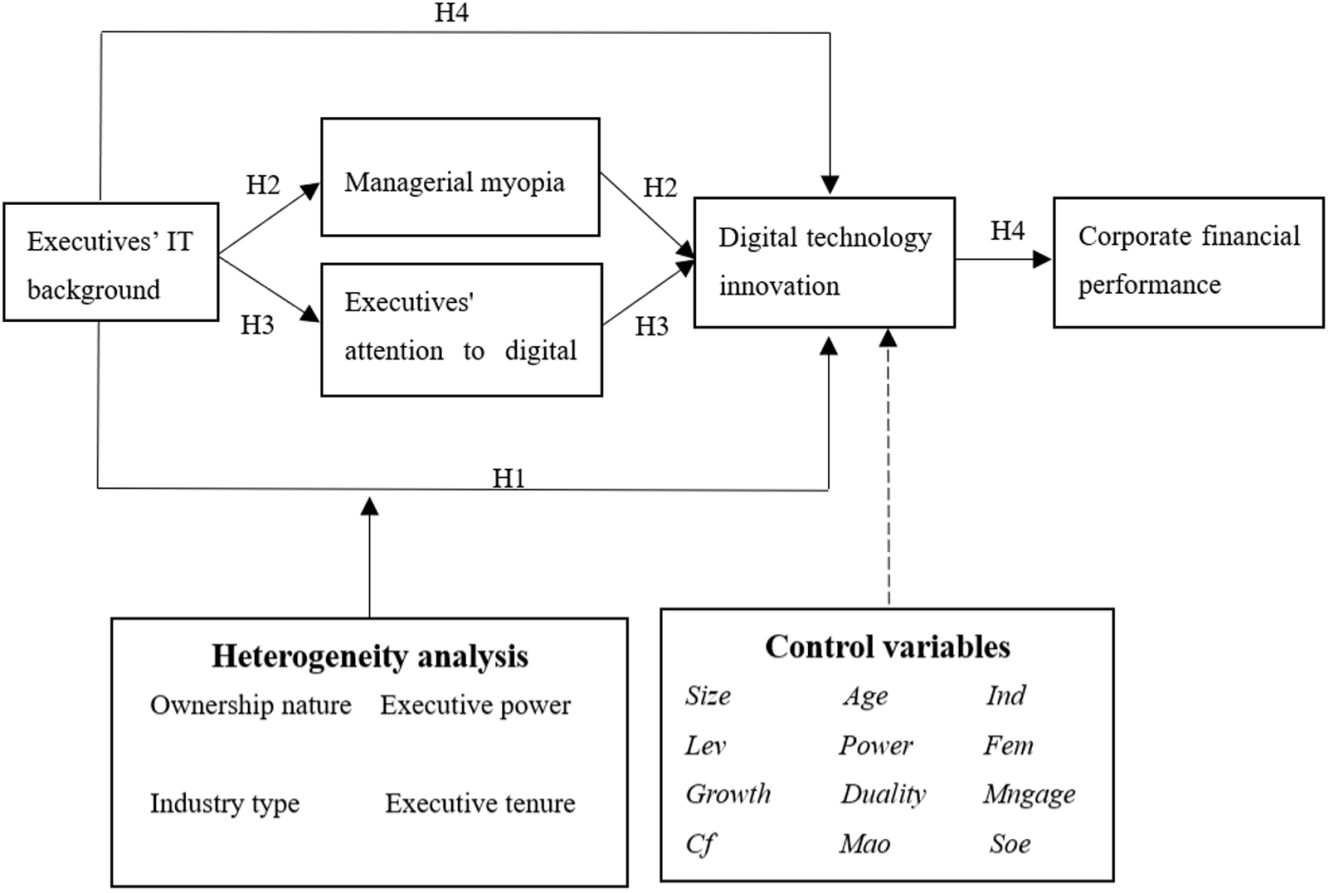
Theoretical analysis framework.

In summary, this paper’s theoretical model is constructed, shown in Fig 1.

## 3. Methods and data

### 3.1 Model settings

#### 3.1.1 Panel benchmark regression model.

Combined with the previous theoretical analysis, this paper sets up the following measurement model to verify hypothesis H1:


$${DiPatent}_{i,t}=\beta_{0}+\beta_{1}{IT}_{i,t}+\beta_{n}{Controls}_{it}+\sum ID+\sum Year+\sum Pro+\varepsilon_{i,t}$$ (1)

In this model, DiPatent represents digital technology innovation. IT represents executives’ IT background. Controls represent the control variables. β is the coefficient, i stands for the firm, t is the year. ID, Year and Pro are industry, year and province fixed effects, respectively, and $\varepsilon_{i,t}$ is a random disturbance term.

#### 3.1.2 Mechanism testing model.

To test hypotheses H2 and H3, this study established a mediation variable model.


$$M_{i,t}=\alpha_{0}+\alpha_{1}{IT}_{i,t}+\alpha_{n}Controls+\sum ID+\sum Year+\sum Pro+\varepsilon_{i,t}$$ (2)

$${DiPatent}_{i,t}=\gamma_{0}+\gamma_{1}{IT}_{i,t}+{\gamma_{2}M}_{i,t}+\gamma_{n}Controls+\sum ID+\sum Year+\sum Pro+\varepsilon_{i,t}$$ (3)

In these models, M represents various mechanism variables, including managerial myopia and executives’ attention to digital technology. a,γ is the coefficient, i stands for the firm, t is the year. ID, Year and Pro are industry, year and province fixed effects, respectively, and $\varepsilon_{i,t}$ is a random disturbance term.

### 3.2 Sample selection and data sources

The sample for this study consists of Chinese A-share listed companies from 2011 to 2021. Exclusions were made for financial and insurance industry listed companies, companies with asset-liability ratios below 0 and above 1, companies with operating losses for two consecutive years, known as special treatment (ST), companies with operating losses for three consecutive years, known as delisting warning (ST*), companies that have stopped any trading, cleared the price, and are waiting for the stock to be delisted (PT), and companies with missing variable observations. These resulted in 22,227 observations. To address extreme values, we winsorize all continuous variables at the 1% level in both upper and lower tails. The sample data for digital technology patents that measure digital technology innovation, originating from the IncoPat Global Patent Database (IncoPat), include details such as company names, types of patents, patent application dates, patent numbers, and IPC codes. Executives’ IT backgrounds and other corporate financial data were sourced from the China Stock Exchange Market and Accounting Research Database (CSMAR).

### 3.3 Definition of variables

Dependent variables: corporate digital technology innovation (DiPatent). Several scholars have attempted to measure digital technology innovation. Liu et al. (2023) [14] assessed corporate digital technology innovation by counting patent applications in digital information transmission. Yang (2022) [66] used textual analysis of patent applications, identifying digital patents for listed companies based on five critical technology keywords under fundamental technology and technology application. This paper builds on previous research, incorporating the Statistical Classification of Digital Economy and Its Core Industries (2021) and the Reference Relationship Table between the International Patent Classification and the National Economic Industry Classification (2018) from the National Bureau of Statistics. This method aligns national economic industry codes with IPC group codes to more precisely identify digital technology innovations at the patent level. Results are matched with corporate patents’ IPC classification numbers. From these matches, we calculate the annual totals for digital technology patent applications (Digiinno), digital technology invention patent applications (Digiinva), and digital technology utility model patent applications (Digiuma) for listed companies.

Independent variable: Executives’ IT background (IT). According to Masli et al. (2016) [9], senior executives are board members and senior management. Assessing executives’ IT backgrounds involves analyzing their educational and professional experiences in information technology. Educationally, it examines whether executives hold computer science, information science, the Internet, and big data degrees or have completed relevant coursework. Professionally, this includes their experience in information technology, systems development, the Internet, cloud computing, and positions in related departments such as IT affairs, network services, and application software. Executives are considered to have an IT background if they possess the above educational or professional qualifications. Using these criteria, we manually compile the number of executives with IT backgrounds from resumes disclosed in the annual reports of listed companies. This figure is compared with the total executive count to evaluate the prevalence of IT expertise within the executive team.

Mechanism variables: Including two mechanistic variables. (1) Managerial myopia (Myopia). Drawing on Brochet et al. (2015) [67], we used Python software to identify 42 short-term-oriented words in listed companies’ MD&A, such as “within the year,” “as soon as possible,” “latest,” “difficulty,” “inflation pressure,” and “upward pressure.” The proportion of these short-term-oriented words to the total word count in MD&A was used as an indicator of managerial myopia. A higher value of the Myopia variable indicates more severe myopic behavior by the management. (2) Executives’ attention to digital technology (Attn). Drawing on Bendig et al. (2023) [64], we used Python software to identify 175 digitalization-related words in the listed company’ MD&A, such as “artificial intelligence technology,” “big data technology,” “cloud computing technology,” “blockchain technology,” “digital technology application,” “internet business models,” “intelligent manufacturing,” and “modern information systems.” The proportion of digitalization-related words to the total word count in the MD&A indicated executives’ attention to digital technology. A higher Attn value signifies greater management emphasis on information technology.

Control Variables: This study utilizes twelve control variables closely associated with digital technology innovation. The Size variable is defined by the logarithmic value of the total year-end assets. The Lev variable represents the ratio of total year-end liabilities to assets. The Growth variable is calculated as the ratio of the year-over-year change in revenue to the previous year’s revenue. The Cf variable is the net operating cash flow ratio to total assets. The Age variable is the logarithm of the years since the company’s establishment. The Power variable is calculated as the ratio of the total shareholdings of the second to fifth largest shareholders to those of the largest shareholder. The Duality variable is assigned a value of 1 if the Chairman and CEO are the same individual; otherwise, it is 0. The Mao variable is the ratio of management’s shareholdings to the total shares. The Ind variable is the ratio of independent directors to the total board members. The Fem variable is calculated as the ratio of females to total executives. The Mngage variable is defined by the logarithm of the average age of executives, incremented by one. The Soe variable is assigned a value of 1 for state-owned or controlled enterprises; otherwise, it is 0. The specific measurement methods for all variables in this study are listed in Table 1.

**Table 1:** Variable definition table.

Variable Type	Variable Name	Variable Description
Dependent variables	Digiinno	Natural logarithm of (number of digital patent applications +1)
	Digiinva	Natural logarithm of (number of digital invention patent applications +1)
	Digiuma	Natural logarithm of (number of digital utility model invention patent applications +1)
Independent variables	IT	The proportion of executives with an information technology background relative to the total number of executives.
Intermediate variables	Myopia	The ratio of short-term oriented words to the total word count in the MD&A.
	Attn	The ratio of terms related to digital technology attention to the total word count in the MD&A.
Control variables	Size	Natural logarithm of total assets
	Lev	Total liability/ Total asset
	Growth	Operating income growth rate
	Cf	Net operating cash flow/ Total asset
	Age	Natural logarithm of the years since the firm's establishment
	Power	The total shareholdings of the second to fifth largest shareholders/The total shareholdings of the largest shareholder
	Duality	If the roles of Chairman and General Manager are held by the same individual, it is 1; otherwise, it is 0
	Mao	The total number of management's shareholdings/ The total number of company shares
	Ind	The total number of independent members/ The total number of board members.
	Fem	The total number of female executives/ The total number of executives
	Mngage	Natural logarithm of the average age of executives
	Soe	If the company is state-owned or state-controlled, it is 1; otherwise, it is 0

## 4. Analysis of empirical results

### 4.1 Descriptive statistics analysis

Table 2 shows the results of the descriptive statistical analysis. The mean value of corporate digital patent applications (Digiinno) is 1.130, with a minimum of 0 and a maximum of 8.512. Overall, firms’ performance in digital technology innovation is relatively weak. The range between the maximum and minimum values indicates significant differences in digital technology innovation capabilities among companies. The mean value of executives’ IT background (IT) is 0.094, suggesting a relatively low proportion of executives with IT backgrounds.

**Table 2:** Descriptive statistical analysis.

Variable	obs	Mean	std	min	max
Digiinno	22227	1.130	1.393	0	8.512
Digiinva	22227	0.835	1.236	0	8.412
Digiuma	22227	0.693	1.066	0	6.770
IT	22227	0.094	0.146	0	1
Size	22227	22.186	1.380	17.641	28.636
Lev	22227	0.404	0.200	0.008	0.997
Growth	22227	0.340	13.109	−0.997	187.371
Cf	22227	0.047	0.072	−1.937	0.664
Age	22227	2.849	0.345	1.098	4.158
Power	22227	0.790	0.648	0.004	4.000
Duality	22227	0.308	0.462	0	1.000
Mao	†Table_Caption22227	16.650	21.313	0	100.000
Ind	22227	37.694	5.640	0	80.000
Fem	22227	18.444	11.041	0	72.220
Mngage	22227	3.912	0.066	3.600	4.157
Soe	22227	0.317	0.465	0	1
Myopia	22227	0.206	0.139	0	2.033
Attn	22227	1.557	1.485	0	6.380

### 4.2 Benchmark regression

Table 3 reports the analysis results of the impact of executives’ IT backgrounds on corporate digital technology innovation. Column (1) shows that without controlling for regional fixed effects, the coefficient of IT is 0.779, which is significant at the 1% level. Column (4) shows that after controlling for regional fixed effects, the coefficient of IT is 0.822, which is significant at the 1% level. This indicates that executives’ IT backgrounds significantly promote corporate digital technology innovation, supporting Hypothesis H1. In Column (5), when digital invention patents are used as the dependent variable, the coefficient of IT is 0.876. In Column (6), when digital utility model patents are used as the dependent variable, the coefficient of IT is 0.282, both of which are significant at the 1% level. This suggests that executives’ IT backgrounds have a more pronounced effect on digital invention patents compared to digital utility model patents.

**Table 3:** Benchmark regression results.

Variable	(1)	(2)	(3)	(4)	(5)	(6)
	Digiinno	Digiinva	Digiuma	Digiinno	Digiinva	Digiuma
IT	0.779***	0.857***	0.223***	0.822***	0.876***	0.282***
	(0.082)	(0.078)	(0.061)	(0.082)	(0.079)	(0.061)
Size	0.365***	0.340***	0.244***	0.364***	0.336***	0.248***
	(0.010)	(0.010)	(0.008)	(0.010)	(0.010)	(0.008)
Lev	−0.309***	−0.330***	−0.037	−0.325***	−0.326***	−0.077**
	(0.050)	(0.044)	(0.039)	(0.050)	(0.044)	(0.039)
Growth	−0.001**	−0.001***	−0.001**	−0.001**	−0.001**	−0.001**
	(0.001)	(0.000)	(0.000)	(0.000)	(0.000)	(0.000)
Ct	0.295***	0.178*	0.362***	0.208*	0.117	0.273***
	(0.111)	(0.098)	(0.088)	(0.110)	(0.098)	(0.087)
Age	−0.031	−0.008	0.010	−0.047*	−0.016	−0.011
	(0.028)	(0.025)	(0.023)	(0.028)	(0.025)	(0.023)
Power	−0.010	0.009	−0.038***	−0.015	0.004	−0.042***
	(0.013)	(0.012)	(0.010)	(0.013)	(0.012)	(0.010)
Duality	0.060***	0.058***	0.036**	0.050***	0.047***	0.030**
	(0.018)	(0.016)	(0.015)	(0.018)	(0.016)	(0.014)
Mao	0.004***	0.003***	0.003***	0.003***	0.003***	0.003***
	(0.000)	(0.000)	(0.000)	(0.000)	(0.000)	(0.000)
Ind	−0.003**	−0.001	−0.002*	−0.003**	−0.002	−0.002
	(0.001)	(0.001)	(0.001)	(0.001)	(0.001)	(0.001)
Fem	−0.003***	−0.002***	−0.002***	−0.002***	−0.002***	−0.002***
	(0.001)	(0.001)	(0.001)	(0.001)	(0.001)	(0.000)
Mngage	−0.545***	−0.333***	−0.715***	−0.311**	−0.169	−0.492***
	(0.144)	(0.127)	(0.114)	(0.145)	(0.128)	(0.114)
Soe	0.116***	0.144***	0.036**	0.131***	0.158***	0.049***
	(0.023)	(0.021)	(0.018)	(0.023)	(0.021)	(0.018)
ID	YES	YES	YES	YES	YES	YES
Year	YES	YES	YES	YES	YES	YES
Pro	NO	NO	NO	YES	YES	YES
Constant	−4.678***	−5.373***	−1.899***	−5.501***	−5.890***	−2.806***
	(0.568)	(0.505)	(0.447)	(0.574)	(0.513)	(0.449)
N	22227	22227	22227	22227	22227	22227
Adjust-R^2^	0.332	0.319	0.285	0.343	0.329	0.301

Note: Figures in brackets are robust standard errors; ^*^ p <0.1, ^**^ p <0.05, ^***^ p <0.01.

### 4.3 Robustness tests

Considering the possible problems of endogeneity, specific industries and regions, different variable measurements, study periods, and economic models in the regression results, this paper conducts the following robustness tests.

#### 4.3.1 Endogeneity test.

Endogeneity issues may influence the conclusions of this study. This study employs the Heckman two-stage method to construct a treatment effects model for empirical testing. For the selection of instrumental variables, this study utilizes the nationwide academic evaluation organized by the Ministry of Education of China in 2017. The evaluation lists universities associated with the key disciplines “0812 Computer Science and Technology” and “0835 Software Engineering.” In regions with listed companies, the number of universities offering these disciplines (NumUniv) is the instrumental variable for executives’ IT backgrounds (IT). Generally, university graduates seek employment locally or in neighboring regions due to the higher recognition of local or nearby universities. Additionally, firms are more likely to hire graduates from local universities due to lower recruitment costs. Therefore, the number of universities offering IT-related disciplines positively correlates with the likelihood of executives having IT backgrounds. At the same time, the number of universities offering IT-related disciplines does not directly affect corporate digital technology innovation, satisfying the exogeneity condition of the instrumental variable.

Table 4 reports the results of the Heckman two-stage test. In Column (1), the coefficient of NumUniv is 0.007, which is significant at the 1% level. The results indicate that the number of universities offering information technology disciplines in the region positively correlates with corporate executives with IT backgrounds. In columns (2) to (4), the IT coefficients are positive and significant at the 1% level. These findings suggest that the analysis results are consistent with the baseline regression after controlling for sample selection bias.

**Table 4:** Heckman two-stage test.

Variable	(1)	(2)	(3)	(4)
	IT	Digiinno	Digiinva	Digiuma
IT		0.234***	0.390***	0.066***
		(0.015)	(0.018)	(0.015)
IMR		−0.138	0.128	0.268**
		(0.100)	(0.116)	(0.105)
NumUniv	0.007***			
	(0.079)			
Controls	YES	YES	YES	YES
ID	YES	YES	YES	YES
Year	YES	YES	YES	YES
Pro	YES	YES	YES	YES
N	22227	22227	22227	22227

Note: Figures in brackets are robust standard errors; ^*^ p <0.1, ^**^ p <0.05, ^***^ p <0.01.

#### 4.3.2 Accounting for the effects of specific industries and regions.

To control the influence of specific industries, we excluded samples from the information technology sector. The results in column (1) of Table 5 show that the coefficient for IT is 0.966, which is significant at the 1% level. Firms in economically developed regions are generally more inclined to adopt digital transformation. To control the influence of specific regions, we excluded samples from Beijing, Shanghai, Guangzhou, and Shenzhen. The results in column (2) of Table 5 indicate that the coefficient for IT is 0.830, which is significant at the 1% level. These findings suggest that the analysis remains consistent with the baseline regression after excluding the effects of specific industries and regions.

**Table 5:** Robustness tests.

Variable	(1) Exclude specific industries	(2) Exclude specific regions	(3) Change the independent variable	(4) Change the dependent variable	(5) Exclude specific sample ranges	(6) The Tobit model
IT	0.966***	0.830***	0.145***	0.092***	0.819***	2.369***
	(0.113)	(0.112)	(0.017)	(0.037)	(0.099)	(0.095)
Controls	YES	YES	YES	YES	YES	YES
ID	YES	YES	YES	YES	YES	YES
Year	YES	YES	YES	YES	YES	YES
Pro	YES	YES	YES	YES	YES	YES
Constant	−4.285***	−5.731***	−5.496***	0.264**	−5.655***	−92.463***
	(0.607)	(0.670)	(0.576)	(0.104)	(0.683)	(10.512)
N	17299	15897	22227	22227	16553	22227
Adjust-R^2^	0.300	0.331	0.341	0.218	0.355	

Note: Figures in brackets are robust standard errors; ^*^ p <0.1, ^**^ p <0.05, ^***^ p <0.01.

#### 4.3.3 Change the independent variable and the dependent variable.

If a top management team member of a listed company has an information technology background, the variable is assigned a value of 1; otherwise, it is assigned a value of 0, serving as a proxy variable for IT. Column (3) of Table 5 shows that the IT coefficient is 0.145, which is significant at the 1% level. The proportion of digital technology patent applications to total patent applications is used as a proxy variable for Digiinno. Column (4) of Table 5 shows that the IT coefficient is 0.092, which is significant at the 1% level. The above results indicate that after changing the measurement methods of the independent and dependent variables, the analysis results are consistent with the baseline regression.

#### 4.3.4 Exclude specific sample ranges.

Due to the significant impact of the COVID-19 pandemic on the external macro environment, this study excluded data from 2020 and 2021. Column (5) of Table 5 shows that the IT coefficient is 0.819, which is significant at the 1% level. This result is consistent with the baseline regression findings and further validates the research hypothesis.

#### 4.3.5 Change the econometric model.

Given the left-censoring characteristics of the data on corporate digital technology innovation, this study employed the Tobit model for analysis. Column (6) of Table 5 shows that the IT coefficient is 2.369, which is significant at the 1% level. Analysis with the Tobit model aligns with baseline regression results.

## 5. Analysis of empirical results

### 5.1 Mechanism analysis

Table 6 reports the analysis results of the mechanisms of managerial myopia. In columns (1), (3), and (5), the IT coefficient is −0.055, which is significant at the 1% level, indicating that executives with an IT background can mitigate managerial myopia. In column (2), the Myopia coefficient is −0.118, significant at the 10% level, and the IT coefficient is 1.674, significant at the 1% level, with the Sobel test confirming the presence of a mediating effect. These findings indicate that executives’ IT background effectively promotes digital technological innovation by alleviating managerial myopia, thereby validating hypothesis H2. In column (4), the Myopia coefficient is −0.147, significant at the 5% level, and the IT coefficient is 1.853, significant at the 1% level, with the Sobel test supporting a mediating effect. In column (6), the Myopia coefficient is −0.014, which is insignificant, and the Sobel test reveals no significant mediating effect. These results suggest that executives’ IT backgrounds significantly promote digital invention patents by mitigating managerial myopia compared to digital utility model patents.

**Table 6:** Mechanism test for managerial myopia.

Variable	(1)	(2)	(3)	(4)	(5)	(6)
	Myopia	Digiinno	Myopia	Digiinva	Myopia	Digiuma
IT	−0.055***	1.674***	−0.055***	1.853***	−0.055***	0.285***
	(0.006)	(0.063)	(0.006)	(0.056)	(0.006)	(0.061)
Myopia		−0.118*		−0.147**		−0.014
		(0.067)		(0.059)		(0.006)
Controls	YES	YES	YES	YES	YES	YES
ID	YES	YES	YES	YES	YES	YES
Year	YES	YES	YES	YES	YES	YES
Pro	YES	YES	YES	YES	YES	YES
Constant	9.115***	−53.232***	9.115***	−50.493***	9.115***	−0.827
	(0.661)	(6.639)	(0.661)	(5.804)	(0.661)	(5.232)
N	22227	22227	22227	22227	22227	22227
Adjust-R^2^	0.102	0.105	0.102	0.131	0.102	0.051
Sobel Z	1.718*		2.413**		0.258	

Note: Figures in brackets are robust standard errors; ^*^ p <0.1, ^**^ p <0.05, ^***^ p <0.01.

Table 7 reports the results of the mechanism of attention to digital technology. In columns (1), (3), and (5), the IT coefficient is 1.934, which is significant at the 1% level, indicating that executives’ IT background significantly enhances attention to digital technology. In column (2), the coefficients of Attn and IT are significantly positive at the 1% level, and the Sobel test confirms the presence of a mediating effect. These results suggest that executives’ IT background effectively promotes corporate digital technology innovation by enhancing attention to digital technology, thus confirming Hypothesis H3. In column (4), the coefficient of IT is 0.608; in column (6), the coefficient of IT is 0.185, both significant at the 1% level, and the Sobel test supports the presence of the mediating effect. These results indicate that, compared to digital utility model patents, executives’ IT backgrounds significantly promote digital invention patents through enhanced attention to digital technology.

**Table 7:** Mechanism test for executives’ attention to digital technology.

Variable	(1)	(2)	(3)	(4)	(5)	(6)
	Attn	Digiinno	Attn	Digiinva	Attn	Digiuma
IT	1.934***	0.562***	1.934***	0.608***	1.934***	0.185***
	(0.073)	(0.082)	(0.073)	(0.078)	(0.073)	(0.061)
Attn		0.134***		0.139***		0.051***
		(0.008)		(0.007)		(0.006)
Controls	YES	YES	YES	YES	YES	YES
ID	YES	YES	YES	YES	YES	YES
Year	YES	YES	YES	YES	YES	YES
Pro	YES	YES	YES	YES	YES	YES
Constant	0.662	−5.590***	0.662	−5.981***	0.662	−2.839***
	(0.510)	(0.570)	(0.510)	(0.509)	(0.510)	(0.447)
N	22227	22227	22227	22227	22227	22227
Adjust-R^2^	0.536	0.353	0.536	0.342	0.536	0.304
Sobel Z	26.555***		29.674***		14.713***	

Note: Figures in brackets are robust standard errors; ^*^ p <0.1, ^**^ p <0.05, ^***^ p <0.01.

### 5.2 Heterogeneity analysis

According to prior analysis, executives with an information technology background significantly positively impact corporate digital technology innovation across the entire sample. However, because different types of companies have varying demands for digital technology innovation, the influence of executives’ information technology background on corporate digital technology innovation depends on the company’s organizational characteristics and the power status of the executives. To further analyze this heterogeneity, the sample was categorized into four aspects: ownership nature, industry type, executive power, and executive tenure, and then regrouped for regression analysis. The grouped regression analysis aims to thoroughly investigate the impact of executives’ information technology background on corporate digital technology innovation under different organizational characteristics, providing a reference for companies to formulate personalized digital strategies.

#### 5.2.1 Ownership nature.

Companies with different ownership natures face distinct management contexts, which may cause the impact of executives’ information technology backgrounds on digital technology innovation to vary. Strategically, state-owned enterprises have a unique relationship with the government, positioning them in a pivotal role in its digitalization strategy. State-owned enterprises may pursue digital technology innovation to better align with government directives. With China’s emphasis on corporate digital technology innovation, state-owned enterprises often shoulder greater social responsibilities, making it easier to incorporate government-promoted digital technology innovation concepts into their decision-making processes. Against the national strategy to promote digital technology innovation, executives with information technology backgrounds in state-owned enterprises can play a pivotal role by leveraging their educational backgrounds and professional experience to consciously and purposefully promote digital technology innovation in their companies. From a resource perspective, state-owned enterprises find it easier than non-state-owned enterprises to obtain bank credit financing. This enables state-owned enterprises to secure more substantial funding support for high-risk, high-investment digital technology innovation. Executives with information technology backgrounds can thus promote digital technology innovation without being overly constrained by financing limitations, allowing their information technology expertise to be utilized more effectively.

Table 8 reports the regression results for state-owned enterprises (SOEs) and non-state-owned enterprises (NSOEs). In column (1), the IT coefficient for the SOE sample is 1.057; in column (2), the IT coefficient for the NSOE sample is 0.685; both coefficients are significant at the 1% level, and the IT coefficient for SOEs is higher than that for NSOEs. The results suggest that the impact of executives’ IT background on digital technological innovation is more pronounced in SOEs, which may be related to the close relationship between SOEs and the government, making SOEs more responsive to the government’s digital innovation strategies.

**Table 8:** Heterogeneity test of ownership nature and industry type.

Variable	(1)	(2)	(3)	(4)
	State-owned enterprises	Non-state-owned enterprises	Information industry	Non-information industry
IT	1.057***	0.685***	0.534***	0.966***
	(0.184)	(0.090)	(0.112)	(0.113)
Controls	YES	YES	YES	YES
ID	YES	YES	YES	YES
Year	YES	YES	YES	YES
Pro	YES	YES	YES	YES
Constant	0.636	−6.836***	−10.628***	−4.285***
	(1.326)	(0.674)	(1.410)	(0.607)
N	7067	15160	4928	17299
Adjust-R^2^	0.410	0.327	0.257	0.300

Note: Figures in brackets are robust standard errors; ^*^ p <0.1, ^**^ p <0.05, ^***^ p <0.01.

#### 5.2.2 Industry type.

While the information industry is driven primarily by digital technologies, they are not central in non-information industries. Adopting digital technologies like artificial intelligence and blockchain can transform business models in non-information industries, facilitating integration with complex ecosystems and comprehensive product and service upgrades. Consequently, digital technology innovation could yield more excellent marginal contributions in non-information industries. Additionally, digital innovation activities in non-information industries are less frequent and pose more significant challenges. As a result, these industries attract more capital market attention, and executives with IT backgrounds could better drive digital technology innovation.

Table 8 reports the regression results for the information industry and non-information industries. In column (3), the IT coefficient for the information industry sample is 0.534; in column (4), the IT coefficient for the non-information industry sample is 0.966; both coefficients are significant at the 1% level, with the IT coefficient for non-information industries exceeding that of the information industry. The results indicate that executives’ IT backgrounds impact digital technology innovation more pronounced in non-information industries. This may be due to the significant challenges faced by non-information industries in applying and integrating digital technologies, where executives with IT backgrounds can leverage their skills to overcome these challenges, thereby driving digital technology innovation.

#### 5.2.3 Executive power.

Power plays a crucial role in resource allocation, influencing corporate investment strategies. High costs and risks of digital technology innovation often cause significant management disagreements over investment decisions. Executives with IT backgrounds wield enhanced decision-making authority in resource allocation when they hold substantial power, supporting digital technology innovation. Additionally, high-power executives with extensive IT knowledge can lead digital technology innovation initiatives and encourage employee participation, advancing digital technology innovation strategies. This approach reduces innovation failure risk and maximizes the utilization of executives’ intentions and expertise. The study employs the IT executives’ compensation ratio to the executive team’s total compensation as a power indicator. Samples are categorized into high-power and low-power groups for regression analysis based on the median industry power indicators.

Table 9 reports the regression results for the high-power and low-power groups. In column (1), the IT coefficient for the high-power group is 0.747, significant at the 1% level; in column (2), the IT coefficient for the low-power group is 0.616, significant at the 5% level, and the IT coefficient for the low-power group is lower than that for the high-power group. The findings suggest that executives with IT backgrounds significantly impact digital technology innovation when they hold more significant power.

**Table 9:** Heterogeneity test of executive power and tenure.

Variable	(1)	(2)	(3)	(4)
	High power	Low power	Longer tenure	Shorter tenure
IT	0.747***	0.616**	0.754***	0.529***
	(0.163)	(0.090)	(0.153)	(0.138)
Controls	YES	YES	YES	YES
ID	YES	YES	YES	YES
Year	YES	YES	YES	YES
Pro	YES	YES	YES	YES
Constant	−3.379**	−6.939***	−7.426***	−3.058***
	(1.359)	(1.040)	(1.305)	(1.077)
N	4813	7199	5053	6959
Adjust-R^2^	0.372	0.338	0.408	0.303

Note: Figures in brackets are robust standard errors; ^*^ p <0.1, ^**^ p <0.05, ^***^ p <0.01.

#### 5.2.4 Executive tenure.

The extended cycles of digital technology innovation suggest that the tenure of executives with IT backgrounds could significantly affect a company’s innovation level. Executives with shorter tenures may prioritize short-term benefits over long-term, high-risk digital technology innovation projects. Digital technology innovation demands that executives understand corporate operations and business models to direct innovation efforts and integrate technology smoothly with business functions. Achieving high-level research and development results in digital technology innovation requires time and effort. This demands persistent enthusiasm, resilience from R&D personnel, and firm conviction and executive determination. Motivated executives with IT backgrounds and longer tenures are more likely to mitigate moral hazards and more effectively implement innovation strategies. This study measures executive tenure by the average tenure of IT-background executives, categorizes samples by industry median tenure, and performs grouped regression analysis comparing longer to shorter tenures.

Table 9 reports the regression results for executives with longer and shorter tenures. In column (3), the IT coefficient for executives with longer tenures is 0.754; in column (4), the IT coefficient for executives with shorter tenures is 0.529, both of which are significant at the 1% level, and the IT coefficient for the longer tenure group is higher than that for the shorter tenure group. The findings suggest that executives with IT backgrounds have a more significant impact on digital technology innovation when they have longer tenure in the firm.

### 5.3 Economic consequences analysis

This study further explores the economic impact of executives’ IT backgrounds on corporate digital technology innovation.This article uses industry-adjusted net profit margins of total assets to assess corporate financial performance. Table 10 reports the analysis results of the economic impact. In columns (1) and (4), the IT coefficient is significantly positive, at least at the 5% level, indicating that executives’ IT backgrounds can improve the company’s financial performance. In columns (2) and (5), the IT coefficient is significantly positive, at least at the 1% level, suggesting that executives’ IT backgrounds drive digital technology innovation, consistent with the baseline regression results. In columns (3) and (6), the Digiinno and IT coefficients are significantly positive, and Sobel tests further indicate the presence of a mediation effect. These results suggest that executives’ IT backgrounds enhance corporate financial performance by promoting digital technology innovation, thus validating hypothesis H4.

**Table 10:** Economic consequences analysis of financial performance.

Variable	(1)	(2)	(3)	(4)	(5)	(6)
	Roa_t_	Digiinno_t_	Roa_t_	Roa_t_+_1_	Digiinno_t_+_1_	Roa_t_+_1_
IT	0.019***	0.822***	0.018***	0.014**	0.925***	0.010*
	(0.004)	(0.082)	(0.004)	(0.006)	(0.089)	(0.006)
Digiinno			0.001***			0.004***
			(0.000)			(0.000)
Controls	YES	YES	YES	YES	YES	YES
ID	YES	YES	YES	YES	YES	YES
Year	YES	YES	YES	YES	YES	YES
Pro	YES	YES	YES	YES	YES	YES
Constant	−0.208***	−5.501***	−0.202***	−0.263***	−4.425***	−0.246***
	(0.043)	(0.574)	(0.043)	(0.040)	(0.633)	(0.040)
N	22227	22227	22227	19068	19068	19068
Adjust-R^2^	0.266	0.343	0.266	0.109	0.347	0.112
Sobel Z	2.044**			1.983**		

Note: Figures in brackets are robust standard errors; ^*^ p <0.1, ^**^ p <0.05, ^***^ p <0.01.

## 6. Conclusions and suggestions

### 6.1 Conclusions

This paper summarizes the impact of executives’ IT backgrounds on corporate digital technology innovation through theoretical analysis. We examine the role of executives’ IT backgrounds in influencing digital technology innovation by using data from Chinese publicly listed companies from 2011 to 2021. The findings indicate that executives’ IT backgrounds significantly enhance digital technology innovation, particularly in digital technology invention patents, which is even more pronounced. Remains robust after a series of robustness checks. Mechanism tests reveal that managerial myopia and executives’ attention to digital technology play significant mediating roles in the influence of executives’ IT backgrounds on digital technology innovation. Further analysis shows that the enhancing effect of executives’ IT backgrounds on digital technology innovation is more significant when the executives are from state-owned and non-IT industries, have longer tenures, and possess greater power. Additionally, the study finds that executives’ IT backgrounds can improve corporate financial performance by promoting digital technology innovation.

### 6.2 Suggestions

This paper enhances our theoretical understanding of how executives’ IT backgrounds influence corporate digital technology innovation. It expands the literature on the economic effects of executives’ IT backgrounds and the factors impacting digital technology innovation in firms. Additionally, the results can help companies harness the advantages of IT talent through positive incentives, advance digital technology innovation and transformation, and provide helpful policy implications.

First, the government should enhance efforts to develop digital technology talent, thereby creating a reserve that supports the high-quality development of China’s digital economy. The government must acknowledge the critical role of IT professionals in driving corporate digital technology innovation, especially within executive management, where a strong leadership team can often dictate the success or failure of such innovations. Currently, there is a widespread challenge of digital technology talent shortages across companies. Governments, businesses, universities, and research institutions must deepen their collaboration, integrating practical and educational resources more effectively to fill vacancies in technical and managerial roles. They should collaboratively develop training models and innovate cooperative mechanisms for digital talent, ensuring alignment between talent supply and demand.

Second, it is crucial to increase the proportion of IT executives in senior management and refine corporate innovation incentives. Companies must prioritize and enhance non-salary incentives for IT talent to advance digital technology innovation strategies and boost innovation performance. Firms should actively recruit IT professionals with domain expertise to bolster their management teams’ digital technology innovation capacity. Additionally, providing strong incentives to IT-skilled staff will enhance their motivation and innovation efficiency.

Third, encouraging executives with an information technology background to participate actively in corporate governance is essential for optimizing governance mechanisms. Many firms prioritize short-term profitability over long-term sustainable development, undermining the momentum for digital technology innovation within the company. Only if management includes a suitable proportion of IT talent and grants them adequate authority and extended tenures can these professionals be genuinely motivated to contribute to the governance structure and support the development and implementation of long-term digital technology innovation strategies.

## 7. Contribution, limitations and future directions

The contributions of this paper are reflected in three main aspects. First, from a research perspective, the paper focuses on the IT background of executives, examining how those with IT education or professional experience influence corporate digital technological innovation. This approach distinguishes itself from studies that concentrate on specific executive positions or analyze the general characteristics of all executives about digital technological innovation. Second, in terms of methodology, the study uses digital technology patents to measure digital technological innovation and applies panel regression, instrumental variable methods, and Tobit models to explore the causal relationship between executives’ IT backgrounds and corporate digital technological innovation. Finally, the paper addresses the content of the research by employing text analysis to uncover managerial short-sightedness and executives’ focus on digital technology. It also uses mediation models to uncover how IT background executives facilitate digital technological innovation, providing additional evidence for the imprinting theory in explaining digital innovation.

However, this study has certain limitations due to data resource constraints. First, this study is based solely on evidence from Chinese micro-enterprises. It does not involve cross-national comparisons, making it difficult to evaluate the universal role of executives’ IT backgrounds in fostering digital technological innovation. Second, certain factors that may influence corporate digital technological innovation, such as the financing environment of the firm and the innovation environment of its location, were not included in the analysis. Finally, digital technological innovation is measured by the number of patent applications, which may not fully capture a firm’s digital technological innovation capabilities.

Therefore, this study can be further explored from the following perspectives. First, future research could examine the cross-national differences in the impact of executives’ IT backgrounds on digital technology innovation. Second, it would be valuable to analyze whether firms’ financing environments and local innovation ecosystems moderate the influence of executives’ IT backgrounds on digital technology innovation. Additionally, future studies could evaluate firms’ digital technology innovation capabilities by investigating the market share of their innovative products and the extent to which consumers or businesses adopt these technologies.

## Supporting information

S1 Data Executives’ IT background and corporate digital technology innovation. (ZIP)
